# Michael Carteighe

**Published:** 1910-06-11

**Authors:** 


The death occurred on May 30 of Michael Carteighe, at the age of 68 years. Mr. Carteighe was president of the Pharmaceutical Society of Great Britain for fourteen years (1882-1896), and was the best-known personality in British pharmacy. He first became a member of the Council in 1866, and was closely associated with the passing of the Pharmacy Act, 1868, the Act which restricted the sale of scheduled poisons to registered chemists and druggists, and placed the education and examination of pharmacists on a regular basis. Mr. Carteighe was born in 1841, and received his scientific training at the School of Pharmacy and at University College, London. He was one of the founders of the Institute of Chemistry, of which body he was for many years a vice-president; he was also for several years a vice-president of the Society of Arts.

